# Apatinib and Ginsenoside-Rb1 Synergetically Control the Growth of Hypopharyngeal Carcinoma Cells

**DOI:** 10.1155/2022/3833489

**Published:** 2022-01-13

**Authors:** YanWei Li, Feng He, Yu Zhang, ZhanYu Pan

**Affiliations:** ^1^Academy of Medical Engineering and Translational Medicine, Tianjin University, Tianjin 300192, China; ^2^Tianjin Key Laboratory of Brain Science and Neural Engineering, Tianjin University, Tianjin 300192, China; ^3^Department of Integrative Oncology, Tianjin Medical University Cancer Institute and Hospital and Key Laboratory of Cancer Prevention and Therapy, Tianjin 300060, China

## Abstract

**Background:**

Apatinib is an anticancer drug known to inhibit the vascular endothelial growth factor receptor-2 (VEGFR-2) through regulating tyrosine kinases. Drug resistance and reduced activity in various cancers is the matter of great concern; thus, researchers opt to use combination of the two or more drugs. So far, its gynergetic anticancer role with a traditional Chinese drug Ginsenoside-Rb1 (G-Rb1) has not been studied in cancers including hypopharyngeal carcinoma.

**Objective:**

The current study is aimed at investigating the anticancer synergetic effects of G-Rb1 and apatinib in hypopharyngeal carcinoma.

**Methods:**

The synergetic effects of both drugs on cell proliferation, wound healing and cell migration, and cell apoptosis were studied in hypopharyngeal carcinoma cells. Furthermore, the xenograft rat model was generated, and tumor inhibition was monitored after treating rats with both drugs as mono- and combination therapy. In addition, protein expression and localization were performed by western blotting and immunofluorescent staining, respectively.

**Results:**

The analyses of the data showed that combination therapy of apatinib and G-Rb1 significantly inhibited the proliferation, migration, and wound healing capability of hypopharyngeal carcinoma cells. Moreover, the glycolysis rate of the cells in the combination therapy (apatinib and G-Rb1) group was significantly decreased as compared to that in the monotherapy group or no treatment group, suggesting that the glycolysis inhibition led to the inhibition of tumor growth. Moreover, the combination therapy on xenograft rats dramatically reduced the tumor size. Furthermore, combination therapy also exhibited an increased count of CD3^+^ and CD4^+^ T cells, as well as the ratio between CD4^+^ and CD8^+^ T cells.

**Conclusion:**

Interestingly, a combination of apatinib and G-Rb1 induced more tumor cell apoptosis and reduced cell proliferation than the individual drug treatment and promote antitumor immunity by enhancing immunomodulatory molecules. Thus, we believe that this study could serve as a valuable platform to assess the synergetic anticancer effects of the herbal as well as synthetic medicines.

## 1. Introduction

The advanced hypopharyngeal carcinoma is usually being treated by a combination of radiotherapy and chemotherapy, showing an overall five-year survival rate between 58.6% and 83% [1–3]. However, the recurrence possibility of the treatment is around 7%-13% in residual tumor and 15%-58% in advanced stage tumor [4]. It is highly believed that the drug resistance could be the main reason for the failure in obtaining desirable treatment outcomes [2, 5]. Hence, new treatment strategies and medicines are needed to cope with the growing concerns of worst survival rate. Therefore, huge attention has been paid to treating tumors with new natural or synthetic drugs. As a selective inhibitor of vascular endothelial growth factor receptor-2 (VEGFR-2) tyrosine kinase, apatinib has antiangiogenic and antitumor effects simultaneously [6] and was thus used in treatment plan of various cancers [7, 8]. However, it has been well established that standalone apatinib therapy does not significantly inhibit the growth of nasopharyngeal carcinoma [9]. On the other hand, herbal medicines are also widely used as traditional medicines to treat different types of cancers including hypopharyngeal carcinoma recently [10]. Such as ginsenoside Rb1 is an effective ingredient of ancient Chinese herbal medicine demonstrating antitumor and antiangiogenic effects [11]. However, ginsenoside has shown antitumor activity against hypopharyngeal cancer cells by targeting the HIF-1*α*-GLUT1 pathway [12] and also enhance the level of T-lymphocytes [13, 14].

Therefore, using apatinib and ginsenoside as combination therapy may not only inhibit the tumor growth and angiogenesis but also stimulate the immune system. Thus, providing a better treatment option for the hypopharyngeal carcinoma. In the current study, for the first time, we aim to investigate the synergetic anticancer effects of apatinib and ginsenoside Rb1 on human and rats' hypopharyngeal carcinoma cell lines as well as in xenograft rat model. Overall, the results of this study could be very useful for treating tumors especially human hypopharyngeal carcinoma.

## 2. Materials and Methods

### 2.1. Cell Culture and Animals

The human hypopharyngeal carcinoma cell line FADU and Rca-b (rat) cells were purchased from the American Type Culture Collection (ATCC Manassas, VA, USA) and cultured in Dulbecco's modified Eagle's medium (DMEM) supplemented with 10% fetal bovine serum (FBS), in a 5% CO_2_ humidified incubator at 37°C. This study was approved by the Ethical Committee of Tianjin University, and all the experiments on rats were conducted in accordance with the guidelines set by the Animal Ethics Committee of Tianjin University. A total of 20 male Sprague-Dawley (SD) rats (age: 3 months, body weight: 350 ± 10 g) were purchased from Animal Center of Tianjin Medical University (Tianjin, China) and fed with purified water and a commercial stock diet and kept in a room equipped with an air conditioner to maintain temperature at 20-26°C.

### 2.2. Cell Viability Assay

The cytotoxicity of both drugs on FUDA and Rca-b cells was determined using a Cell Counting Kit-8 (CCK-8) assay kit (Beyotime Biotechnology Co. Ltd., China). Briefly, FUDA and Rca-b cells in the logarithmic growth period were collected and dispensed into 96-well cell culture plates (5000 cells/well), treated with the different concentrations of apatinib and G-Rb1. Following incubation for 24, 48, or 72 h, 10 *μ*l of CCK-8 was added to each well, and 2 h later, the optical density (OD) at 450 nm was measured by using a microplate reader (Bio-Rad Laboratories, Richmond, CA, USA).

### 2.3. Colony Formation Assay

Cells treated with apatinib at different concentrations for 48 h were seeded in 6-well plates at a density of 3000 cells per well. The cells were cultured with fresh medium containing different concentrations of single or combined drugs and allowed to grow for 2 weeks, then fixed with 4% paraformaldehyde, and stained with 1% Crystal violet (Beyotime Biotechnology Co. Ltd., China). The colonies were counted in each group, and results of combination therapy were compared with no treatment as well as monotherapy groups.

### 2.4. Immunofluorescence

Rca-b cells were seeded on glass coverslips in six-well plates for 24 h. The medium was then replaced by fresh DMEM media supplemented with 10% FBS and apatinib, ginsenoside Rb1 or apatinib+ginsenoside Rb1. After incubation for 24 h, the cells were fixed with 4% paraformaldehyde for 30 min at RT, permeabilized with 0.3% Triton X-100 for 10 min, and blocked with goat serum (Solarbio, Beijing, China) for 30 min at RT. Then cells were incubated with anti-ki67 antibodies (1 : 500, ab92742, Abcam) overnight at 4°C. The following day after washing with TBST, the coverslips were incubated with fluorescent secondary antibodies (Proteintech, Wuhan, China) for 2 h at RT. The nuclei were counterstained with 4′, 6-diamidino-2-phenylindole (DAPI) and mounted and imaged under the ZEISS LSM880 confocal microscope.

### 2.5. Cell Apoptosis Assay

Cells were cultured in a six-well plates and treated with mono as well as combination of apatinib and ginsenoside Rb1 with different concentrations (apatinib: 2.5-40 *μ*M; G-Rb1: 5-80 *μ*M). After culturing cells for 72 h, Annexin V-FITC and propidium iodide (KeyGEN Biotech, Jiangsu, China) stains were added to cells and population of apoptotic cells was analyzed by using flow cytometer (BD, Franklin Lake, NJ, USA) and FlowJo software, version 7.6.2 (TreeStar, Ashland, OR, USA).

### 2.6. Western Blot Analysis

Cells were lysed in protein lysis buffer (50 mM Tris-Cl (pH = 8.0), 150 mM NaCl, 5 m ethylenediaminetetraacetic acid (EDTA), 1% NP-40, and 1 mM phenylmethylsulfonyl fluoride). Equal amounts of protein samples of different treatments were performed via electrophoresis (SDS-PAGE) and transformed to nitrocellulose (Amersham Pharmacia Biotech, Piscataway, NJ, USA), subsequently incubated in blocking buffer (5% skimmed milk, 0.1% Tween-20 in 20 mmol/l Tris-buffered saline) at room temperature for 1 h. Then, membranes were incubated with primary antibodies prepared in 5% TBST skimmed milk overnight at 4°C slow shaking. The next day, they were washed and incubated with HRP-conjugated secondary antibody. Blots were developed by enhanced chemiluminescence (ECL) (Amersham Pharmacia Biotech, Piscataway, NJ, USA).

### 2.7. Xenograft Rats and Treatment Groups

The 5 × 10^6^ cells/ml of Rca-b cells were diluted in sterilized phosphate-buffered saline (PBS), and 0.2 ml of cell suspension was administered through a hypodermic needle to the right axilla of each rat. After 7 days of cell administration, solid tumors were examined. Rats were randomly divided into four groups (control group, apatinib group, G-Rb1 group, and apatinib+G-Rb1 group); each group contain 5 rats. Normal saline was injected for 18 days in the control group through the caudal vein, whereas an oral dose 50 mg/kg of apatinib and G-Rb1 was given orally to the apatinib group and ginsenoside Rb1 groups, respectively, for 18 days, and the same treatment protocol was adopted for the rats in the apatinib+G-Rb1 group.

### 2.8. Rate of Tumor Inhibition and Immune Organ Index

The rate of tumor inhibition was evaluated to estimate the efficacy of different concentrations and combinations of the drugs on the tumors. After 18 days of drugs administration, the rats were anesthetized by diethyl-ether and killed; whole body weight was calculated in each group; upon dissection, the weight of the tumor, spleen, and thymus was calculated in each group. The rate of tumor inhibition was calculated by the following formula: rate of tumor inhibition (%) = (1–average weight of tumor in the group treated by drug/average weight of tumor in the NS group) × 100%.

### 2.9. Glycolysis Assay

Cells were seeded in a 6-well plate and maintained overnight in DMEM. The next day, the culture medium was replaced with the fresh complete medium with different drugs for an additional 48 h. The glucose uptake by cells was measured using a Glucose Uptake Colorimetric Assay kit (BioVision, USA) according to the manufacturer's protocol. For lactate measurement, the cells were cultured in DMEM without phenol red (Hyclone, USA) but supplemented with single or both drugs for 48 h, then culture medium was collected, and lactate levels were determined using a Lactate Colorimetric Assay kit (BioVision, USA).

### 2.10. Flow Cytometry for Detecting Antigens (CD3^+^, CD4^+^, and CD8^+^)

After 12 hours of the last doses of drugs, the rats were anesthetized by diethyl ether and blood samples from each group were obtained in the containers containing anticoagulant. Blood samples were treated with hemolysin to make lymphocyte suspensions of 1 × 10^6^/ml. Each suspension was added with Alexa Fluor® 488 anti-rat CD3^+^, Alexa Fluor® 594 anti-rat CD4^+^, and Alexa Fluor® 647 anti-rat CD8^+^ (eBioscience, San Jose, CA, USA) and protected from light. Then, flow cytometry analysis was performed on FACSCalibur flow cytometer (Franklin Lakes and Becton Dickinson, NJ, USA) to evaluate the differential levels of antigens (or CD3^+^, CD4^+^, and CD8^+^) in each group. The data obtained were analyzed by Cell Quest software (San Jose, BD Biosciences, CA, USA).

### 2.11. Statistical Analysis

All data were presented as the mean ± standard deviation (or SD for short). Student's *t*-test was used to find out the significant differences between the groups. The counting data were expressed as percentage (%) and verified by a chi-square test and denoted as *χ*^2^. *P* value less than 0.05 was considered significant.

## 3. Results

### 3.1. Association between Expression of VEGFR2 (KDR) and Survival of the Patients

**Figure 1:**
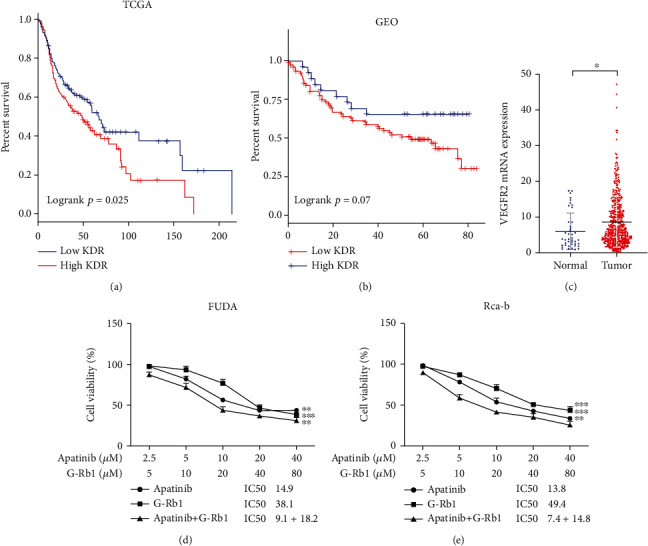
The effect of apatinib and G-Rb1 in hypopharyngeal carcinoma. (a) The effect of VEGFR2 expression (TCGA data) on prognosis. (b) Influence of VEGFR2 expression (GEO database) on prognosis. (c) Relative mRNA expression of VEGFR2 in normal and tumor tissues. Apatinib and G-Rb1 inhibit proliferation of the (d) FUDA cells and the human hypopharyngeal cancer cells (e). Rca-b on rat buccal squamous cells. ^∗^*P* < 0.05, ^∗∗^*P* < 0.01, and ^∗∗∗^*P* < 0.001 vs. control.

Angiogenesis is a complex process regulated by the balance between proangiogenic and antiangiogenic factors. Vascular endothelial growth factor (VEGF) is one of the most important proangiogenic factors. It stimulates angiogenesis by binding to the VEGF receptors (VEGFR1 and VEGFR2) and tyrosine kinases (RTKs) on the cell surface of endothelial cells (EC). The RNA-seq data analysis from different databases (TCGA and GEO database) showed the association of high expression of VEGFR2 with worse prognosis of the cancer patients (Figures 1(a) and 1(b)), while VEGFR2 was found to have significantly higher expression in tumor than the normal tissues (Figure 1(c), *P* < 0.05).

### 3.2. Combination Therapy of G-Rb1 and Apatinib Reduces the Viability of the Cells

**Figure 2:**
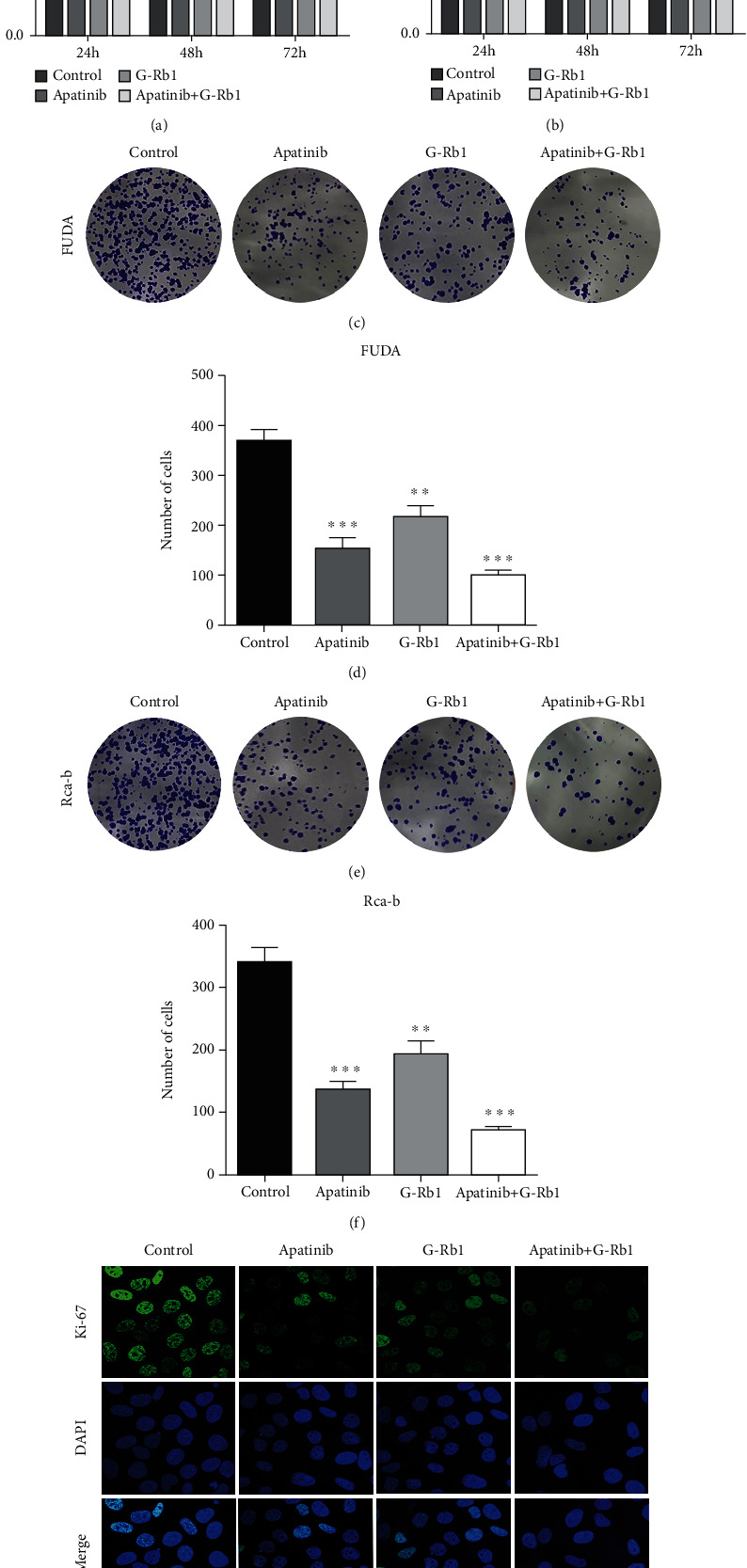
Effects of mono and combination of apatinib and G-Rb1 on FUDA and Rca-b cells. (a, b) Combination of apatinib and G-Rb1 inhibited the proliferation of FUDA and Rca-b at different time points. (c–f) Combination of apatinib and G-Rb1 significantly reduced the colony formation capability of the FUDA and Rca-b cells. (g) The expression of Ki67 significantly affected in combination with apatinib- and G-Rb1-treated FUDA and Rca-b cells. ^∗∗^*P* < 0.01 and^∗∗∗^*P* < 0.001 vs. control.

The analysis of the data revealed that both apatinib and ginsenoside Rb1 inhibit the proliferation of FUDA and Rca-b cells in a dose-dependent manner (Figures 1(d) and 1(e), *P* < 0.05). As IC50 value was designed for single drug therapy, therefore, we studied the combination of the apatinib and G-Rb1 in molar ratio (apatinib: G-Rb1) of 1 : 2 for 24-72 h. As compared with the treatment outcome of individual drugs, the combination treatment strategy exhibited significant inhibitory effects on the cell proliferation (Figures 2(a) and 2(b), *P* < 0.05). Colony formation assay proved that the cotreatment reduced FUDA and Rca-b viability and tumor formation (Figures 2(c)–2(f), *P* < 0.05). Also, the expression of ki-67 in the apatinib+G-Rb1 significantly reduced compared to those in other groups in the Rca-b cells (Figure 2(g)).

### 3.3. Cotreatment of Ginsenoside Rb1 and Apatinib-Affected Cell Functions

**Figure 3:**
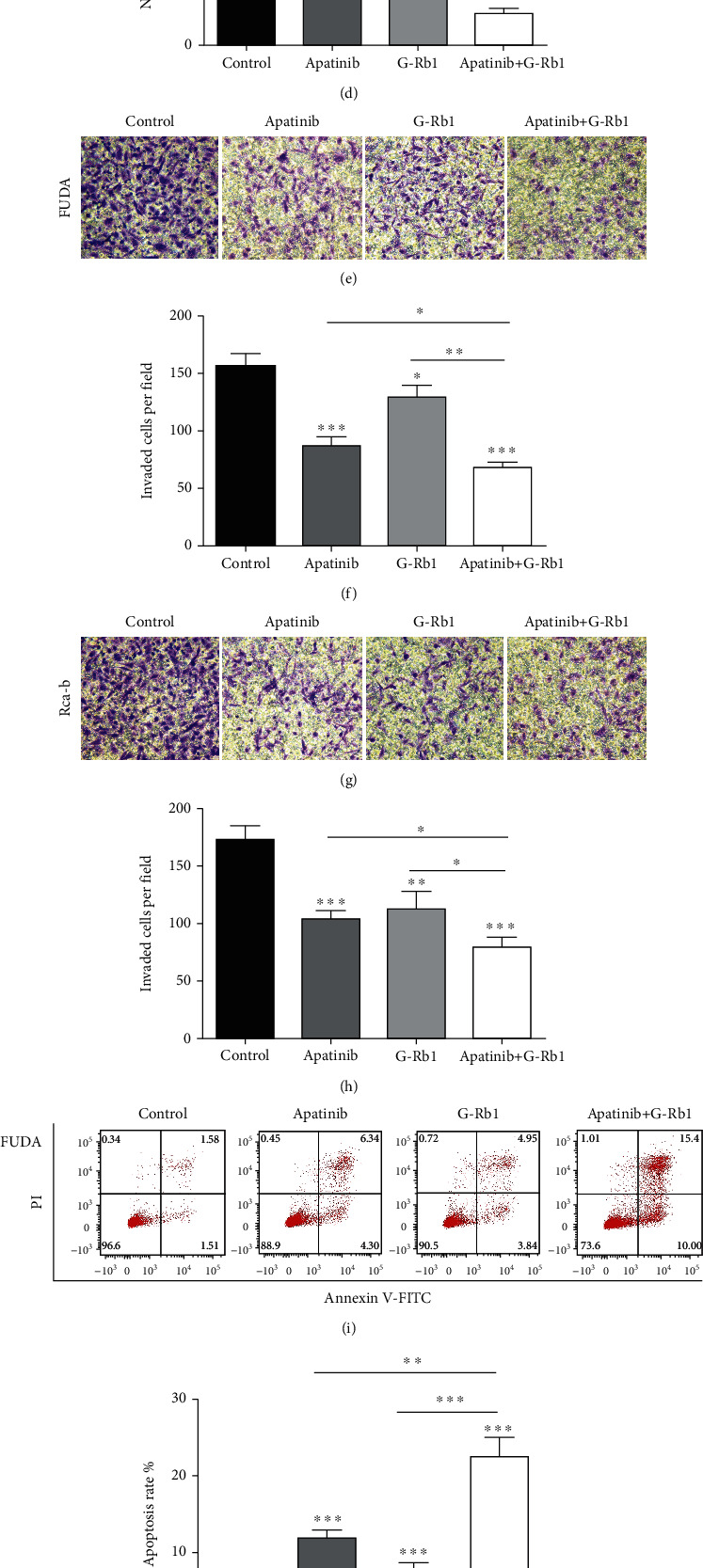
Apatinib and G-Rb1 affect the cell function of FUDA and Rca-b cell. (a–d) Effect of mono and combination of the apatinib and G-Rb1 on the migration. (e–h) Invasion of the FUDA and Rca-b cells. (i–l) The combination therapy of apatinib- and G-Rb1-induced apoptosis in FUDA and Rca-b cells. ^∗^*P* < 0.05,  ^∗∗^*P* < 0.01, and^∗∗∗^*P* < 0.001 vs. control.

Wound healing and transwell migration assays were used to evaluate the migration ability of the FUDA and Rca-b cells. The combination therapy significantly affected the wound healing ability (Figures 3(a)–3(d), *P* < 0.05) and the migration of the FUDA and Rca-b cells as compared with the other treatment groups (Figures 3(e)–3(h), *P* < 0.05). We also examined the cell apoptosis by flow cytometry which revealed that both G-Rb1 and apatinib treatment induced apoptosis in the studied cells, while effect of the combination therapy was significantly higher as compared to the single drug treatment and control (Figures 3(i)–3(l), *P* < 0.01).

### 3.4. Combined Treatment of Apatinib and G-Rb1 Suppresses Glycolysis

**Figure 4:**
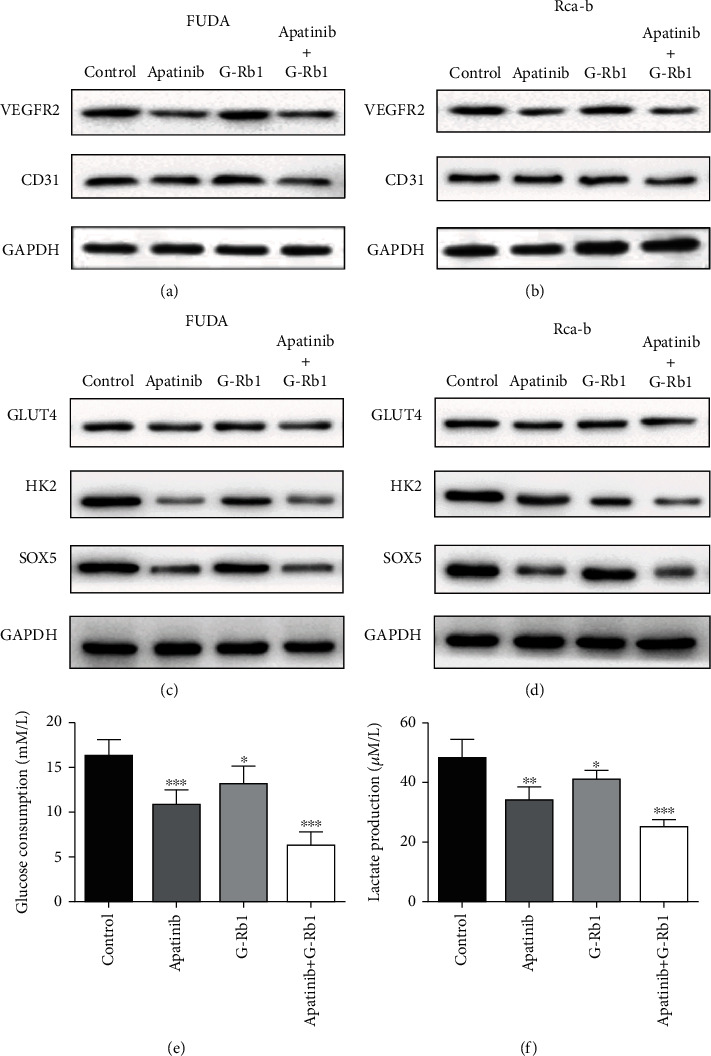
Apatinib and G-Rb1 affect the proteins of angiogenesis and glycolysis signaling pathways of FUDA and Rca-b cells. (a, b) Combination of apatinib and G-Rb1 inhibited VEGFR2 and CD31 expression of FUDA and Rca-b. (c, d) GLUT4, HK2, and SOX5 were downregulated in the combination of apatinib and G-RB1 treated FUDA and Rca-b. The levels of (e) glucose consumption and (f) extracellular lactate production in Rca-b. ^∗^*P* < 0.05,  ^∗∗^*P* < 0.01, and^∗∗∗^*P* < 0.001 vs. control.

To further assess the underlying role of apatinib+G-Rb1 in the altered cell proliferation and functions, we studied the expression of vascular proliferation marker and glycolysis. The protein expression of the studied markers in the apatinib group showed significant reduction than the G-Rb1 group. However, the combination of apatinib+G-Rb1 showed more reduction in the expression of the vascular proliferation markers and signaling pathways (GLUT4, HK2, and SOX5) as compared to other groups in the FUDA and Rca-b cells (Figures 4(a)–4(d)), showing synergetic effect of the both drugs for regulation of the vascular proliferation. In addition, the glucose uptake and lactate production assays in Rca-b cells treated with apatinib, ginsenoside Rb1, or apatinib+G-Rb1 showed significant reduction in glucose uptake and lactate production as compared with the controls in Rca-b cells (Figures 4(e) and 4(f), *P* < 0.05).

### 3.5. Combination Therapy of Apatinib and G-Rb1 Significantly Reduced Tumor Growth

**Figure 5:**
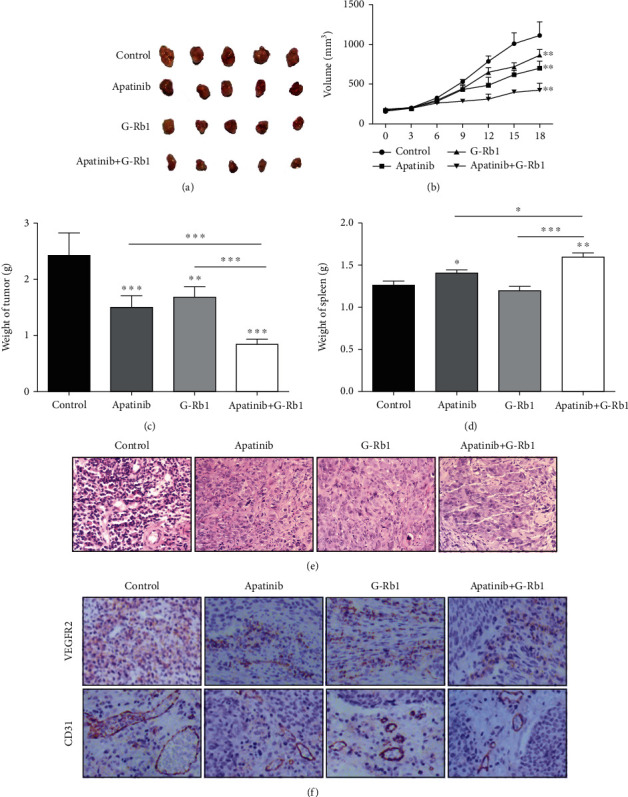
Effects of mono and combination of apatinib and G-Rb1 on the development of Rca-b subcutaneous tumor. (a–c) Combination of apatinib and G-Rb1 inhibits the size, volume, and weight of Rca-b subcutaneous tumors in rats. (d) Spleen weight was significantly increased after apatinib+G-Rb1 treatment. (e) Representative Tumor HE of each group. (f) Effects of apatinib and G-Rb1 on vascular proliferation markers VEGFR2 and CD31 in different groups. ^∗^*P* < 0.05,  ^∗∗^*P* < 0.01, and^∗∗∗^*P* < 0.001 vs. control.

The tumor growth was recorded and evaluated every two days; we noticed that the treatment with apatinib reduced the tumor growth significantly faster than the G-Rb1 therapy while the combined treatment of G-Rb1 and apatinib showed significant reduction in tumor size and weight as compared to the monotherapy and controls. However, no significant change was observed in the total body weight. The tumor inhibition rates in the apatinib group, G-Rb1 group, and apatinib+G-Rb1 group were 37.0%, 29.5%, and 64.8%, respectively, demonstrating that the apatinib+G-Rb1 group exhibited better tumor inhibition (Figures 5(a)–5(c), *P* < 0.05).

### 3.6. Regulation of Spleen In Vivo Tumor Growth Assay

Furthermore, we also found that combination therapy significantly increased the weight of spleen in xenograft rats than the other treatment groups (Figures 5(d), *P* < 0.05). This indicates that combination therapy could potentially regulate the function of immune cells in rats.

### 3.7. Combined Treatment of G-Rb1 and Apatinib Inhibits Angiogenesis In Vivo

The immunohistochemical staining of VEGFR-2 and CD31 in xenograft rat tissues was significantly decreased in combination therapy group (Figures 5(e) and 5(f)). Additionally, the proportion of VEGFR-2-positive and CD31-positive cells in rats in combination therapy was significantly reduced than the control group.

### 3.8. Influence of Ginsenoside Rb1 on T-Lymphocytes in Rats' Peripheral Blood

The number of lymphoid follicle and marginal zone of the spleen's width increased in the combination therapy group than the monotherapy or control groups, but lymphoid follicle diameter remained the same (Table 1). The combination treatment of apatinib+G-Rb1 showed a significant increase in the frequency of CD3^+^ and CD4^+^ cells as well as the ratio between CD4^+^ and CD8^+^ cells in the peripheral blood (*P* < 0.05). However, no significant change in the frequency of CD8^+^ was observed in all groups, whereas the ratio in the apatinib group was slightly higher than that in the control group (Table 2).

### 3.9. Adverse Effects of the G-Rb1 Treatment

Following the G-Rb1 and apatinib treatment, we carefully observed the rats for possible adverse effects; no abnormal change in the daily behavior, ingestion, autonomic activity, hydrophobia, feces, pelage, urine, or short-term allergic reactions were observed in all treatment groups.

## 4. Discussion

Apatinib has been known to act as an antiangiogenic inhibitor and is used as a useful anticancer drug [15]. A recent study regarding apatinib administration on three patients diagnosed with the hypopharyngeal carcinoma, metastatic squamous cell carcinoma of head and neck, and squamous cell carcinoma of the pyriform sinus demonstrated partial reduction in target lesion of tumor size after thirty days of the treatment [16]. The extensive treatment of apatinib in patients with gastric cancer failed to show any significant improvement in the disease condition [17]. However, a HER2-positive GC patients treated with the apatinib showed superior clinical outcomes, suggesting apatinib as a promising anticancer drug for advanced AFP-producing and HER2-positive GC tumors [18]. Additionally, it also failed to show satisfactory improvement in the patients with breast cancer. In short, only a moderate improvement was noted in the patients previously treated with other drugs [19]. Consequently, it has been reported that apatinib could be an effective option for the HER2-positive tumors. Luckily, a female patient with HR-positive and HER2-negative stage of breast cancer showed significant improvement in the symptoms upon low dose of apatinib [20]. However, the details about other additional treatments or clinical history were not provided; hence, efficacy of the apatinib in this case is doubtful. Although it has been studied in combination with other drugs such as paclitaxel and bevacizumab [21, 22], but no study regarding its combination with G-Rb1 is so far reported.

On the other hand, G-Rb1 had shown inhibitory effects in lung cancer [23]. Meanwhile, G-Rb1 also increased the oxidative stress and apoptosis in the rats [24]. Additionally, G-Rb1 was reported to induce the apoptosis and autophagy in the human cervical cancer cells through activating caspase pathways and downregulating PERK and IRE1a [25]. G-Rb1 was known to have beneficial clinical significance in various illness and multiple pathways and have relatively few side effects and thus drawn a great attention of the medical researchers. The data of previous studies revealed that ginsenosides might improve vascular remodeling (VR) caused by vasodilation dysfunction, abnormal vascular structure, and blood pressure [26]. Ginsenoside promotes the expression of IRS-1 in the insulin signaling pathway and played a crucial role in alleviating inflammation and insulin resistance in obesity [27]. G-Rb1 has been studied in combination with berberine diabetic patients which showed amazing results [28]. Collectively, these data showed that G-Rb1 could show more efficiency if tasted in combination with other drugs.

Interestingly, the apatinib monotherapy showed a reasonable efficiency against tumors in several cancers but never been studied in hypopharyngeal carcinoma. It has been well known that apatinib and G-Rb1 individually enhance the immune system which could be useful against tumor [9, 29]. Here, for the first time, we investigated the synergetic effects of the apatinib and G-Rb1 treatment in hypopharyngeal carcinoma. Based on the current findings, we concluded that a combination of the drugs could be a potential therapeutic option for the hypopharyngeal carcinoma and other cancers. Furthermore, our study indicated that G-Rb1 may have vital effects in the reduction of the hypopharyngeal carcinoma in vivo. Previously, apatinib showed useful effects when applied in combination with other drugs such as apatinib combination with CQ-induced apoptosis in ESCC cells through activating IRE-1*α*-AKT-mTOR signaling pathway [30]. Randomized controlled trials of apatinib in combination with SOX showed effective results and acceptable safety profile as a neoadjuvant treatment for locally advanced GC [31].

Otto Warburg demonstrated that tumors preferentially engage in glycolysis even in the presence of oxygen. Several studies have demonstrated that altered glycolysis in HNSCC is associated with the activation of hypoxia-inducible factor-1 (HIF-1), transketolase-like protein 1 (TKTL1), mutations in tumor suppressor gene p53, and overexpression of the glucose transporters-1 (GLUT-1) [32]. However, there was no study about glycolysis in hypopharyngeal carcinoma. As a tyrosine kinase inhibitor, apatinib inhibits glycolysis by suppressing the VEGFR2/AKT1/SOX5/GLUT4 signaling pathway in ovarian cancer cells [33]. As a bioactive component obtained from panax ginseng, ginsenoside Rh4 suppressed glycolysis and the expression of PD-L1 in esophageal cancer [34]. Therefore, it is worth studying the role of apatinib and ginsenoside in tumors with high degree of malignancy, such as hypopharyngeal cancer.

In addition, our findings revealed that the combination therapy could synergetically inhibit the proliferation of hypopharyngeal carcinoma cell lines. This combination therapy largely reduced the tumor growth including volume and weight, and it also indirectly showed antiangiogenesis and immunomodulatory effects [35, 36]. Moreover, these results not only serve as a base for the further development of more effective antitumor therapies but also established a platform for evaluating effects of herbal and synthetic drugs. Ginsenoside Rb1 showed appropriate immunomodulatory and antitumor activities, while combination of apatinib and G-Rb1 significantly increased the proportion of CD3^+^ and CD4^+^ T cells and improved the ratio of CD4^+^/CD8^+^ in the thymus and spleen. Our results are in agreement with previous studies that G-Rb1 treatment in rats increases the oxidative stress and apoptosis along with the increased number of the CD4^+^ and CD8^+^ cells and the levels of cytokines [32, 37]. It has been shown that different components of the ginseng including GE5, GE50, and Rb1 reduce the inflammation by reducing the TNF-*α* and IL-6 cytokine levels in cancer cachexia mice, thereby improving the symptoms of cancer cachexia [38]. G-Rb1 promotes the antitumor immunity through maintaining gut microflora and ameliorates gut mucositis by modulating Nrf2 and NF-*κ*B pathways [39]. A type of ginseng called black ginseng also showed a role in immunity by reducing ROS and NO followed by inflammation. It has also been shown to have antioxidant effects through ER mechanism [40].

In conclusion, the current study using apatinib and G-Rb1 as combination therapy significantly affected the cell proliferation, migration, and wound healing ability and enhanced the apoptosis in hypopharyngeal carcinoma cells. In vivo analysis showed that the combination therapy effectively reduced the tumor growth probably by inhibiting rate of glycolysis. Moreover, combination therapy also showed an increased count of CD3^+^ and CD4^+^ T cells and the ratio between CD4^+^ and CD8^+^ T cells. Overall survival was also increased in the combination therapy group. Thus, we confirm that combination of apatinib and G-Rb1 could be useful treatment regimen for the hypopharyngeal carcinoma.

## Figures and Tables

**Table 1:** The number and width of lymphoid follicles and marginal zones in the spleen using microscopy.

Group	Lymphoid follicle number	Lymphoid follicle diameter	Marginal zone width
Control	4.20 ± 0.74	27.92 ± 5.75	2.21 ± 1.35
Apatinib	6.31 ± 0.89^a^	25.01 ± 7.01	4.76 ± 1.29^a^
G-Rb1	8.52 ± 0.91a	29.60 ± 6.58	6.71 ± 1.07^a^
Apatinib+G-Rb1	9.93 ± 1.11^bc^	29.74 ± 7.10	7.82 ± 1.54^ac^

Four fields were randomly selected from each section (^a^*P* < 0.05 compared with the control group; ^b^*P* < 0.01 compared with the control group; ^c^*P* < 0.05 compared with the apatinib group).

**Table 2:** Lymphocyte subsets in rats.

Group	CD3	CD4	CD8	CD4/CD8
Control	40.34 ± 1.22	30.00 ± 1.21	28.59 ± 0.89	1.33 ± 0.078
Apatinib	44.21 ± 1.01	31.85 ± 1.08	29.88 ± 1.10	1.39 ± 0.066
G-Rb1	62.57 ± 0.97^b^	39.60 ± 1.72^b^	30.78 ± 1.44	1.58 ± 0.094^a^
Apatinib+G-Rb1	64.54 ± 1.04^bc^	40.36 ± 0.89^bc^	27.59 ± 1.13	1.60 ± 0.084^ac^

^a^ *P* < 0.05 compared with the control group; ^b^*P* < 0.01 compared with the control group; ^c^*P* < 0.05 compared with the apatinib group.

## Data Availability

Data generated through this work could be requested from the corresponding author via reasonable request.
